# Effect of acute exercise and exercise training on the ability of insulin to clear branched-chain amino acids from plasma in obesity and type 2 diabetes

**DOI:** 10.1007/s00125-025-06454-y

**Published:** 2025-05-22

**Authors:** Pauline M. Møller, Rasmus Kjøbsted, Maria H. Petersen, Martin E. de Almeida, Andreas J. T. Pedersen, Jørgen F. P. Wojtaszewski, Kurt Højlund

**Affiliations:** 1 Steno Diabetes Center Odense, Odense University Hospital, Odense, Denmark; 2 Department of Clinical Research, University of Southern Denmark, Odense, Denmark; 3 The August Krogh Section for Molecular Physiology, Department of Nutrition, Exercise and Sports, University of Copenhagen, Copenhagen, Denmark; 4 Department of Sports Science and Clinical Biomechanics, University of Southern Denmark, Odense, Denmark

**Keywords:** Acute exercise, Branched-chain amino acids, Exercise training, Obesity, Type 2 diabetes

## Abstract

**Aims/hypothesis:**

Insulin resistance in obesity and type 2 diabetes is associated with elevated plasma branched-chain amino acid (BCAA) levels. Here, we examined whether the ability of insulin to clear plasma BCAAs and any influence of acute exercise or exercise training on this response are intact in obesity and type 2 diabetes.

**Methods:**

In four case–control studies of participants with type 2 diabetes matched to glucose-tolerant individuals with obesity and lean individuals, who underwent hyperinsulinaemic–euglycaemic clamps, we examined the effect of insulin on plasma BCAAs (studies I–IV), with or without prior acute exercise (60 min, 70% $\dot{V}{\mathrm{O}}_{\mathrm{2max}}$) (study II), and before and after 10 weeks of endurance exercise training (study III) or 8 weeks of high-intensity interval training (study IV).

**Results:**

Insulin sensitivity was reduced in individuals with type 2 diabetes compared with individuals with obesity (study I–IV) and lean individuals (studies I and IV), and in individuals with obesity vs lean individuals (study I) (all *p*<0.05). Exercise training (studies III and IV) increased insulin sensitivity in all groups (all *p*<0.01). Plasma BCAAs were elevated in individuals with type 2 diabetes compared with individuals with obesity (studies I, III and IV) and lean individuals (studies I and IV) (all *p*<0.05). The ability of insulin to reduce plasma BCAAs was significantly attenuated in participants with type 2 diabetes compared with both lean individuals (studies I and IV) and individuals with obesity (studies I, II and IV) (all *p*<0.05). Acute exercise slightly reduced plasma BCAAs in both individuals with type 2 diabetes and individuals with obesity but did not potentiate insulin’s ability to reduce plasma BCAAs (study II). Exercise training had no impact on fasting BCAAs and did not affect insulin’s ability to reduce plasma BCAAs in any group (studies III and IV) or rescue the attenuated insulin suppression of plasma BCAAs in participants with type 2 diabetes.

**Conclusions/interpretation:**

Our results demonstrate that insulin’s ability to suppress plasma BCAAs is impaired in type 2 diabetes but is intact in individuals with obesity. Although acute exercise reduces fasting BCAA levels, neither acute exercise nor exercise training affects insulin’s ability to suppress plasma BCAAs in glucose-tolerant individuals with or without obesity or in individuals with type 2 diabetes.

**Graphical Abstract:**

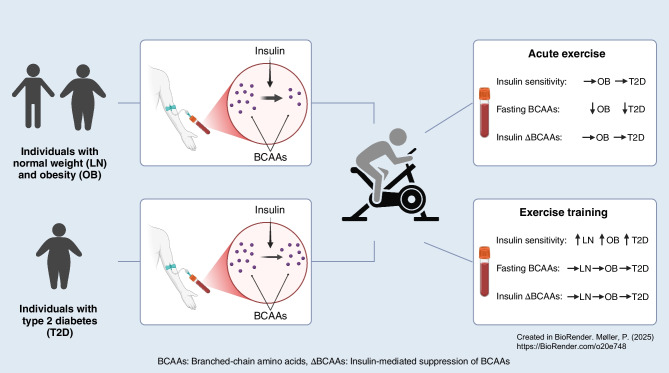

**Supplementary Information:**

The online version contains peer-reviewed but unedited supplementary material available at 10.1007/s00125-025-06454-y.


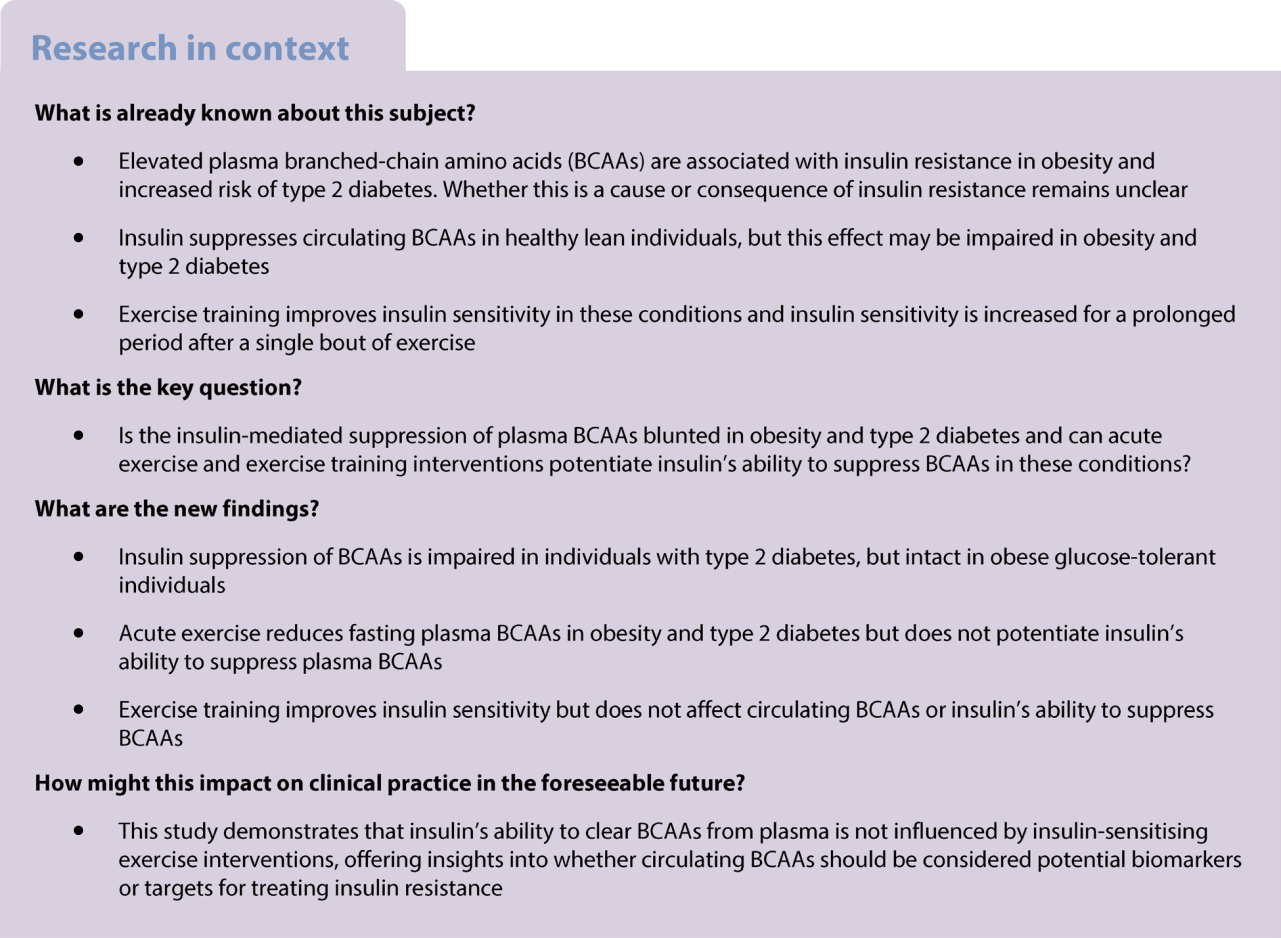


## Introduction

The prevalence of type 2 diabetes has more than tripled over the past two decades, primarily as a consequence of the growing obesity pandemic and sedentary lifestyles [1, 2]. Insulin resistance is the major link coupling obesity to type 2 diabetes and is characterised by dysregulations of multiple processes in key metabolic tissues. These include reduced insulin-stimulated glucose uptake and storage in skeletal muscle, increased lipolysis in adipose tissue and increased hepatic glucose production [3].

Upon ingestion of proteins, insulin stimulates the uptake of amino acids in skeletal muscle [4]. Here, insulin elicits its anabolic effects by enhancing protein synthesis while simultaneously inhibiting proteolysis [4, 5]. Among the circulating essential amino acids are the branched-chain amino acids (BCAAs): leucine, isoleucine and valine. Elevated plasma BCAAs have consistently been associated with obesity and type 2 diabetes [6, 7] and found to predict type 2 diabetes onset [8], but also to be a consequence of insulin resistance [9, 10]. In healthy individuals, insulin reduces plasma BCAAs by ~20–50% [11, 12]. However, in glucose-tolerant individuals with obesity, the ability of insulin to suppress plasma BCAA levels is diminished compared with that of lean individuals [13], and this impaired response is even more pronounced in individuals with type 2 diabetes compared with individuals with obesity [12].

Exercise training has several health benefits in obesity and type 2 diabetes, such as improved insulin sensitivity, cardiorespiratory fitness and body composition, as well as glycaemic management in type 2 diabetes [14]. In skeletal muscle, the adaptations to exercise training include increased abundance of enzymes involved in insulin signalling and glucose metabolism [15] and improved mitochondrial function and content [16, 17]. High-intensity interval training (HIIT) has emerged as an equally effective, or possibly superior, alternative to endurance exercise training with respect to insulin sensitivity, cardiorespiratory fitness and body composition in healthy lean individuals [18], but also in individuals with obesity and type 2 diabetes [19, 20]. Furthermore, insulin sensitivity is increased even after a single bout of exercise [21], for a period up to ~48 h, in both healthy lean individuals [22] and individuals with type 2 diabetes [23]. While exercise training does not seem to change plasma BCAAs in healthy lean, overweight or type 2 diabetic individuals [12, 24, 25], there is evidence that different types of acute exercise reduce plasma BCAAs in healthy lean and overweight individuals [24, 26–32]. Some studies in smaller cohorts suggest that exercise training does not improve the insulin-suppressive effect on plasma BCAAs in overweight/obesity or type 2 diabetes [12, 25]. However, it remains to be clarified whether exercise training and acute exercise potentiate insulin’s ability to suppress plasma BCAAs and to what extent these responses are intact in obesity and type 2 diabetes [11, 24, 25].

In the present study, we investigated whether the ability of insulin to suppress circulating BCAAs is affected in obesity and type 2 diabetes, whether acute exercise and different types of exercise training potentiate insulin action on plasma BCAAs in humans and whether these effects are intact in obesity and type 2 diabetes.

## Methods

### Participants

This is a secondary report of four case–control studies (studies I–IV) in which we originally examined the effects of insulin, acute exercise and exercise training on several parameters in glucose-tolerant individuals with obesity and lean individuals as well as in obese participants with type 2 diabetes [15, 20, 33–35]. Sex was self-reported and in line with their Danish civil registration numbers. Race or ethnicity data were not collected. Further details about eligibility criteria, drop-outs during exercise training and medication are provided in these reports. All the studies included hyperinsulinaemic–euglycaemic clamps (HECs), which were all performed after an overnight fast. Participants were instructed to refrain from strenuous exercise for 48 h before each HEC, and non-insulin glucose-lowering as well as lipid- and blood pressure-lowering medications were withdrawn 1 week prior to the HEC. All studies were conducted at Odense University Hospital and were approved by the Regional Scientific Ethical Committees for Southern Denmark. Informed consent was obtained from all volunteers prior to participation, and the studies were conducted in accordance with the Declaration of Helsinki.

### Study designs

In study I, we examined the effect of insulin on plasma BCAAs in ten participants with obesity and type 2 diabetes group-wise matched by age, sex and BMI to ten glucose-tolerant individuals with obesity and by age and sex to 12 healthy, glucose-tolerant lean individuals (electronic supplementary material [ESM] Table 1). Compared with the original report [34], two additional lean individuals were included as reported previously [35]. The participants underwent a 4 h HEC (insulin infusion rate of 40 mU m^−2^ min^−1^) with tracer glucose ([3-H^3^]-glucose) to assess total glucose disposal rates (GDRs) as a measure of whole-body insulin sensitivity. Blood samples for measurement of plasma BCAAs were collected in the basal state before and 2 h after initiating insulin infusion.

In study II [33], we investigated the effect of prior acute exercise on insulin’s ability to regulate plasma BCAAs in 13 men with obesity and type 2 diabetes matched by age and BMI to 14 glucose-tolerant men with obesity (ESM Table 2). At 1 week prior to the first HEC, the participants underwent exercise tests to determine $\dot{V}{\mathrm{O}}_{\mathrm{2max}}$. All participants underwent two 4 h HECs, one at baseline and one on the exercise day, planned 4–8 weeks apart. The HECs were performed using an insulin infusion rate of 40 mU m^−2^ min^−1^ with tracer glucose ([3-H^3^]-glucose) to assess total GDR. On the exercise day, participants exercised on cycle ergometers for 60 min at an intensity of 70% of $\dot{V}{\mathrm{O}}_{\mathrm{2max}}$, followed by the 4 h post-exercise HEC, initiated 3 h into recovery. The participants continued to be fasting after the exercise bout. Blood samples for measurement of plasma BCAAs were drawn in the basal state before and 60, 90 and 120 min following insulin infusion during the HECs both at baseline and on the exercise day.

In study III [15], we examined how 10 weeks of endurance exercise training affected the effect of insulin on plasma BCAAs in 13 men with obesity and type 2 diabetes matched by age and BMI to 13 glucose-tolerant men with obesity (ESM Table 3). Exercise tests were performed before, midway through and after 10 weeks of training to determine $\dot{V}{\mathrm{O}}_{\mathrm{2peak}}$, as previously described [36]. The training protocol consisted of 4–5 sessions/week of cycling on stationary bikes for 20–35 min, with an average intensity of ~65% of $\dot{V}{\mathrm{O}}_{\mathrm{2max}}$. At 1–2 weeks prior to the start of the training programme and 48 h after its completion, the participants underwent a 3 h HEC (insulin infusion rate of 80 mU m^−2^ min^−1^) with tracer glucose ([3-H^3^]-glucose) to assess total GDR. Blood samples for measurement of plasma BCAAs were drawn in the basal state before and 1 h and 2 h after initiating insulin infusion during the HECs both before and after the training programme.

In study IV [20], we examined the effect of an 8 week supervised HIIT protocol on insulin’s ability to change plasma BCAAs in 15 men with obesity and type 2 diabetes matched by age and BMI to 15 glucose-tolerant men with obesity and by age to 18 glucose-tolerant lean men (ESM Table 4). Before, halfway through and after the HIIT programme, the participants underwent incremental exercise tests to determine $\dot{V}{\mathrm{O}}_{\mathrm{2max}}$, as reported previously [20]. The 8 week HIIT protocol consisted of supervised training on rowing and cycle ergometers (three sessions per week) with increasing blocks (from two to five) of 5×1 min high-intensity intervals (>85% of maximal heart rate) interspersed by 1 min recovery periods and a 4 min break when switching exercise forms. At 1–2 weeks before the HIIT protocol and 48 h after the final $\dot{V}{\mathrm{O}}_{\mathrm{2max}}$ test, the participants underwent a 3 h HEC (insulin infusion rate of 40 mU m^−2^ min^−1^) with tracer glucose to assess total GDR. Blood samples for measurement of plasma BCAAs were drawn in the basal state before and 2 h after starting insulin infusion during the HECs both before and after the HIIT protocol.

In all four studies, the metabolic clearance rate (MCR) of glucose at the end of the clamp was calculated as total GDR divided by the average plasma glucose concentration.

### BCAA assay

The BCAAs, leucine, isoleucine and valine, were measured in basal and insulin-stimulated plasma samples using a commercial enzyme-based assay kit (Abcam, Cambridge, UK) according to the manufacturer’s instructions. Briefly, leucine standards and 20 µl of undiluted plasma were loaded in duplicates, including a set of background controls for each sample, and incubated with enzyme and substrate mix for colorimetric reaction. Absorbance was measured at 450 nm on a Multiscan FC Microplate Photometer (Thermo Fisher Scientific, Waltham, MA, USA). The background reading was subtracted from the measurements, and BCAA concentrations were calculated from the standard curve.

### Statistics

The statistical analyses were performed using GraphPad Prism v.10.0 (Dotmatics, Boston, MA, USA) (downloaded from https://www.graphpad.com/). Unless otherwise stated, tests of statistical significance between groups at basal and insulin-stimulated states were performed using either one-way ANOVA or two-way ANOVA analyses, followed by Šídák's multiple comparisons test as post hoc testing upon significant interactions. Effects of intervention and insulin within exercise studies were evaluated using multiple paired two-way ANOVA tests or mixed models for missing paired samples, with Šídák's multiple comparisons tests. The data are presented as means±SEM, with *p* values below 0.05 considered significant.

## Results

### Clinical and metabolic characteristics

Selected clinical and metabolic characteristics in studies I–IV are given in ESM Tables 1–4. In all studies, all groups were matched on age, and the obese groups with or without type 2 diabetes had similar BMI, fat mass and lean body mass. Fasting plasma glucose and HbA_1c_ were higher in the type 2 diabetes groups compared with the obese and lean groups (all *p*<0.001). Fasting serum insulin was elevated in the type 2 diabetes group vs both the obese and lean groups in study IV (all *p*<0.05), while no differences were observed in studies I, II or III. While baseline $\dot{V}{\mathrm{O}}_{\mathrm{2max}}$ was similar in the type 2 diabetes and obese groups in studies II and III, $\dot{V}{\mathrm{O}}_{\mathrm{2max}}$ was lower in the type 2 diabetes group vs both the obese and lean groups (all *p*<0.001) and in the obese vs lean group (*p*<0.05) in study IV. Insulin sensitivity, measured as insulin-stimulated GDR, was reduced in individuals with type 2 diabetes compared with the glucose-tolerant individuals with obesity (31–48%) and lean individuals (41–58%) in all studies (all *p*<0.05), and in individuals with obesity vs lean individuals (18%) in study I (*p*<0.05). MCR of glucose during euglycaemia at the end of the HEC showed the same differences between the groups within studies I–IV (ESM Tables 1–4).

Endurance training (study III) increased whole-body insulin sensitivity by 17–21% in the type 2 diabetes and obese groups (all *p*<0.01) and HIIT (study IV) increased whole-body insulin sensitivity by 29–42% in all groups (all *p*<0.001), whereas prior acute exercise did not change whole-body insulin sensitivity in individuals with obesity or in individuals with type 2 diabetes (study II). Both exercise training regimens (studies III and IV) increased MCR of glucose at the end of the HEC in all groups (all *p*<0.05), whereas no effect of acute exercise (study II) on MCR of glucose was seen in the type 2 diabetes or obese groups (ESM Tables 1–4). Exercise training (studies III and IV) also increased $\dot{V}{\mathrm{O}}_{\mathrm{2max}}$ in all groups (all *p*<0.01). BMI was slightly reduced in the obese group (*p*<0.001) after endurance training (study III) but did not significantly affect body composition in any of the groups. In contrast, HIIT (study IV) reduced BMI and fat mass and increased lean body mass in all groups (all *p*<0.05). There were no significant differences in these responses between the groups.

### Insulin suppression of plasma BCAAs is blunted in type 2 diabetes

**Fig. 1:**
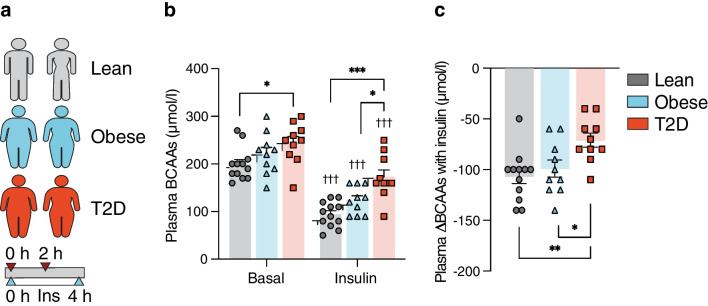
Study I: plasma BCAAs were measured (**a**) before (0 h) and after (2 h) insulin infusion during a 4 h HEC in 12 glucose-tolerant lean participants, ten glucose-tolerant individuals with obesity and ten individuals with type 2 diabetes. (**b**) Plasma BCAAs in the basal and insulin-stimulated states in the lean (grey), obese (blue) and type 2 diabetes (orange) groups. (**c**) Insulin-mediated changes in plasma BCAAs (ΔBCAAs) in the lean (grey), obese (blue) and type 2 diabetes (orange) groups. The data are presented as means ± SEM. *p<0.05, **p<0.01, ***p<0.001 as indicated, ^†††^*p*<0.001 vs basal. Ins, insulin; T2D, type 2 diabetes

In study I, we compared plasma BCAA levels measured before and after 2 h of insulin in individuals with type 2 diabetes, glucose-tolerant individuals with obesity and lean individuals (Fig. 1a). Basal plasma BCAA levels were 1.2-fold higher in individuals with type 2 diabetes compared with lean individuals (244±14 vs 199±10 µmol/l, *p*=0.021), but not individuals with obesity (244±14 vs 221±14 µmol/l, *p*=0.441). There was no difference between individuals with obesity and lean individuals (221±14 vs 199±10 µmol/l, *p*=0.448) (Fig. 1b). Insulin strongly reduced plasma BCAA levels by 44–53% in the lean and obese groups (all *p*<0.001) and by 29% in the type 2 diabetes group (*p*<0.001). In the insulin-stimulated state, plasma BCAA levels were higher in individuals with type 2 diabetes compared with both individuals with obesity (173±15 vs 124±9 µmol/l, *p*=0.015) and lean individuals (173±15 vs 94±8 µmol/l, *p*<0.001), whereas no difference was observed between individuals with obesity and lean individuals (124±9 vs 94±8 µmol/l, *p*=0.192) (Fig. 1b). The insulin-mediated suppression of plasma BCAAs was attenuated by 28–33% in individuals with type 2 diabetes compared with both individuals with obesity (*p*=0.040) and lean individuals (*p*=0.005), while no difference in insulin-mediated suppression of BCAAs was observed between individuals with obesity and lean individuals (*p*=0.745) (Fig. 1c).

### The impact of acute exercise on plasma BCAAs in obesity and type 2 diabetes

**Fig. 2:**
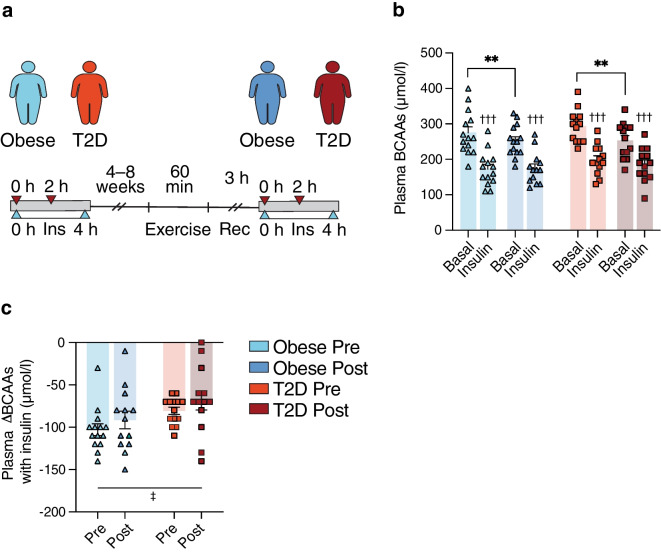
Study II: plasma BCAAs were measured (**a**) before (0 h) and after (2 h) insulin infusion during two 4 h HECs in 14 glucose-tolerant individuals with obesity and 13 individuals with type 2 diabetes performed 4–8 weeks prior to an acute bout of exercise (60 min at 70% of $\dot{V}{\mathrm{O}}_{\mathrm{2max}}$) and again 3 h into recovery. (**b**) Plasma BCAAs prior to exercise in the obese (light blue) and type 2 diabetes (orange) groups and following exercise in the obese (dark blue) and type 2 diabetes (red) groups, both in the basal and insulin-stimulated states. (**c**) Insulin-mediated changes in plasma BCAAs (ΔBCAAs) in the obese (light blue) and type 2 diabetes (orange) groups before exercise (Pre) and in the obese (dark blue) and type 2 diabetes (red) groups following exercise (Post). The data are shown as means ± SEM. **p<0.01 as indicated, ^†††^*p*<0.001 vs basal, ^‡^*p*<0.05 main effect of groups. Ins, insulin; Rec, recovery; T2D, type 2 diabetes

In study II, we evaluated whether prior acute exercise altered insulin’s ability to suppress plasma BCAA levels in individuals with type 2 diabetes and glucose-tolerant individuals with obesity (Fig. 2a). Plasma BCAA levels in the basal and insulin-stimulated states were not different between the two groups, and insulin markedly decreased plasma BCAA levels by 31–37% in both groups (all *p*<0.001), regardless of prior acute exercise (Fig. 2b). This response was preserved when including all insulin-stimulated values (main effect *p*<0.001) (ESM Fig. 1a). Acute exercise caused a reduction in basal plasma BCAA levels in both individuals with obesity (*p*=0.003) and individuals with type 2 diabetes (*p*=0.005). However, prior acute exercise did not change plasma BCAA levels in the insulin-stimulated state in either of the groups compared with the baseline day. The insulin-mediated suppression of plasma BCAAs was reduced in individuals with type 2 diabetes compared with individuals with obesity (main effect *p*=0.017), but with no additional effect of acute exercise (Fig. 2c).

### The effect of endurance exercise training on plasma BCAAs in obesity and type 2 diabetes

**Fig. 3:**
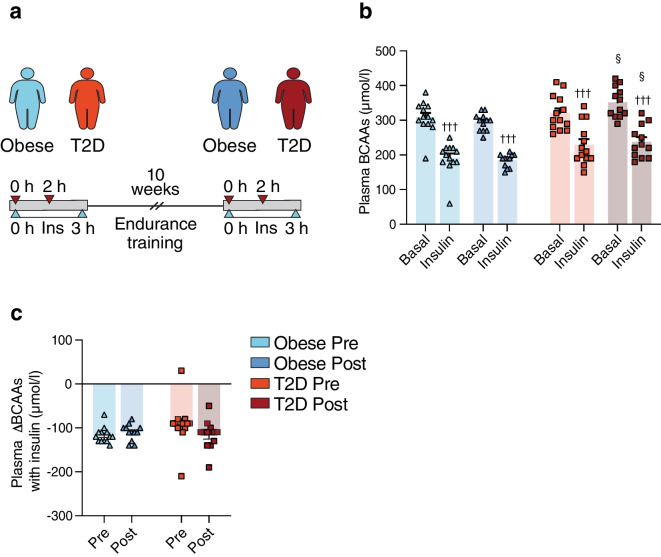
Study III: plasma BCAAs were measured (**a**) before (0 h) and after (2 h) insulin infusion during two 3 h HECs in 13 glucose-tolerant individuals with obesity and 13 individuals with type 2 diabetes performed 1–2 weeks before (Pre) and 48 h after (Post) 10 weeks of endurance exercise training. (**b**) Plasma BCAAs before exercise training in the obese (light blue) and type 2 diabetes (orange) groups and following exercise training in the obese (dark blue) and type 2 diabetes (red) groups, both in the basal and insulin-stimulated states. (**c**) Insulin-mediated changes in plasma BCAAs (ΔBCAAs) in the obese (light blue) and type 2 diabetes (orange) groups before and in the obese (dark blue) and type 2 diabetes (red) groups following exercise training. The data are shown as means ± SEM. ^†††^*p*<0.001 vs basal, ^§^*p*<0.05 vs Obese Post. T2D, type 2 diabetes

In study III, we investigated whether 10 weeks of endurance exercise training affected insulin’s ability to suppress plasma BCAAs in individuals with type 2 diabetes and glucose-tolerant individuals with obesity (Fig. 3a). Prior to exercise training, basal plasma BCAA levels were not different between the groups. However, following exercise training, plasma BCAA levels were higher in individuals with type 2 diabetes compared with individuals with obesity both in the basal (352±13 vs 294±7 µmol/l, *p*=0.011) and the 2 h insulin-stimulated states (238±13 vs 184±6 µmol/l, *p*=0.023) (Fig. 3b), and also when including all plasma BCAAs measured (main effect *p*<0.001) (ESM Fig. 1b). Insulin markedly (~35%) decreased plasma BCAA levels in both groups prior to and following exercise training (all *p*<0.001), and also when including 1 h insulin-stimulated measures (main effect *p*<0.001) (Fig. 3b, ESM Fig. 1b). Basal plasma BCAA levels did not change in response to exercise training in glucose-tolerant individuals with obesity (*p*=0.284), but tended to increase in individuals with type 2 diabetes (352±13 vs 320±14 µmol/l *p*=0.054). Exercise training did not change plasma BCAA levels in the insulin-stimulated states in either group (Fig. 3b). In addition, there were no differences in the insulin-mediated suppression of plasma BCAAs between the groups either before or after exercise training, and exercise training had no impact on insulin’s ability to reduce plasma BCAAs in either group (Fig. 3c).

### The effect of HIIT on plasma BCAAs in obesity and type 2 diabetes

**Fig. 4:**
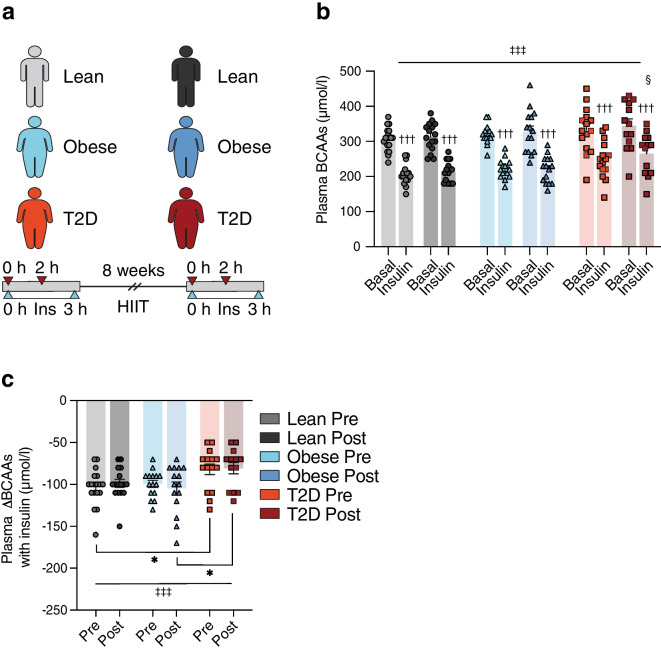
Study IV: plasma BCAAs were measured (**a**) before (0 h) and after (2 h) insulin infusion during two 3 h HECs in 18 glucose-tolerant lean individuals, 15 glucose-tolerant individuals with obesity and 15 individuals with type 2 diabetes performed 1–2 weeks before (Pre) and 48 h after (Post) 8 weeks of HIIT. (**b**) Plasma BCAAs before HIIT in the lean (light grey), obese (light blue) and type 2 diabetes (orange) groups and after HIIT in the lean (dark grey), obese (dark blue) and type 2 diabetes (red) groups, both in the basal and insulin-stimulated states. (**c**) Insulin-mediated changes in plasma BCAAs (ΔBCAAs) in the lean (light grey), obese (light blue) and type 2 diabetes (orange) groups before HIIT and in the lean (dark grey), obese (dark blue) and type 2 diabetes (red) groups after HIIT. The data are shown as means ± SEM. *p<0.05 as indicated, ^†††^*p*<0.001 vs basal, ^‡‡‡^*p*<0.001 main effect of group, ^§^*p*<0.05 vs Lean Post. T2D, type 2 diabetes

In study IV, we examined whether 8 weeks of HIIT changed the insulin-mediated suppression of plasma BCAAs in individuals with type 2 diabetes, glucose-tolerant individuals with obesity and lean individuals (Fig. 4a). Basal plasma BCAAs were not different between the three groups either before or after the HIIT intervention. Insulin significantly decreased plasma BCAAs by 23–33% before and after HIIT in all groups (all *p*<0.001) (Fig. 4b). When considering all measures, fasting and insulin-stimulated, pre and post HIIT, plasma BCAAs were significantly higher in individuals with type 2 diabetes compared with glucose-tolerant individuals with obesity and lean control individuals (main effect *p*<0.001). Additionally, after HIIT, plasma BCAA levels in the insulin-stimulated state were significantly higher in participants with type 2 diabetes compared with lean participants (265±16 vs 214±8 µmol/l, *p*=0.036). However, HIIT did not significantly change plasma BCAA levels in either the basal or insulin-stimulated states in any of the groups. When including all measures before and after HIIT, the insulin-mediated suppression of plasma BCAAs was significantly impaired in individuals with type 2 diabetes compared with lean individuals and individuals with obesity (main effect *p*=0.005) (Fig. 4c). Prior to HIIT, the ability of insulin to suppress plasma BCAAs was reduced in individuals with type 2 diabetes compared with lean individuals (*p*=0.048), but not individuals with obesity (*p*=0.168). Conversely, following the HIIT intervention, insulin-mediated changes in plasma BCAAs were similar in individuals with type 2 diabetes and lean individuals (*p*=0.463), but reduced in individuals with type 2 diabetes compared with glucose-tolerant individuals with obesity (*p*=0.038) (Fig. 4c). HIIT did not change insulin-mediated suppression of BCAAs in any of the groups.

## Discussion

In the present study, we investigated whether insulin’s ability to reduce plasma BCAAs is compromised in obesity and type 2 diabetes. Additionally, we assessed whether acute exercise and exercise training could potentiate insulin action on plasma BCAAs, and whether these responses remain intact in obesity and type 2 diabetes. Our main findings showed that insulin markedly suppressed plasma BCAAs in all examined groups, and that this effect was impaired in type 2 diabetes but not in obesity. Furthermore, we observed that acute exercise reduced plasma BCAAs, and this effect was preserved in individuals with type 2 diabetes. However, acute exercise did not potentiate insulin’s ability to suppress plasma BCAAs. Endurance training did not affect plasma BCAAs in glucose-tolerant individuals but tended to increase fasting BCAA levels in participants with type 2 diabetes, whereas HIIT had no effect on plasma BCAAs in any of the groups. In addition, exercise training did not potentiate the insulin-mediated suppression of plasma BCAAs. These results provide evidence that insulin’s ability to reduce plasma BCAAs is impaired in type 2 diabetes, which may contribute to the higher plasma BCAAs observed in individuals with type 2 diabetes. However, neither acute exercise nor exercise training influence insulin’s ability to suppress plasma BCAAs in humans, despite improved whole-body insulin sensitivity. This suggests that the insulin-sensitising effects of physical activity do not include a reduction in the potential detrimental metabolic consequences of elevated plasma BCAAs.

Elevated plasma BCAAs have long been associated with insulin resistance in obesity and type 2 diabetes and an increased risk of type 2 diabetes [6, 8, 37]. Earlier genome-wide association studies (GWAS) suggested that elevated plasma BCAAs contribute to the risk of type 2 diabetes [38], while more recent reports have provided evidence for a causal role of insulin resistance on plasma BCAAs [9, 10]. In agreement with previous findings, we show that plasma BCAAs are elevated in individuals with type 2 diabetes compared with healthy lean individuals and, although less consistently, compared with glucose-tolerant individuals with obesity. Importantly, we demonstrated that insulin’s ability to suppress plasma BCAAs is impaired in individuals with type 2 diabetes compared with glucose-tolerant individuals with and without obesity in three of our studies. This is consistent with the results in a previous study of men with type 2 diabetes compared with BMI-matched control participants [12], but another study reported similar suppressive effects of insulin on plasma leucine and BCAAs, as well as leucine turnover and oxidation, in normal-weight participants with type 2 diabetes and normal-weight control participants [39]. Moreover, unlike previous observations [13, 40], we were unable to demonstrate impaired insulin-mediated suppression of plasma BCAAs in individuals with obesity. These discrepancies are likely explained by differences in the sample size and sex composition, as well as the degree of insulin sensitivity and glucose tolerance in the examined groups, which are all relatively small. Collectively, our results support the notion that impaired insulin action, rather than obesity per se, may be the primary factor contributing to elevated plasma BCAAs, as observed in the individuals with type 2 diabetes in our study. However, further studies in larger cohorts are needed to fully establish this explanation.

Following acute exercise, circulating BCAA levels are reduced [24, 29], most likely due to increased BCAA uptake and oxidation in skeletal muscle [41]. This effect of acute exercise on plasma BCAAs has been reported to be intact in overweight individuals with dysglycaemia, despite reduced insulin sensitivity and decreased expression of genes involved in BCAA catabolism in muscle [31]. We extend these findings by demonstrating that acute exercise reduces plasma BCAAs in individuals with type 2 diabetes to the same extent as in matched glucose-tolerant individuals with obesity. Moreover, we demonstrate that the insulin-mediated suppression of plasma BCAAs was not potentiated by acute exercise in either individuals with type 2 diabetes or glucose-tolerant individuals with obesity. This is somewhat surprising given the established effects of acute exercise in potentiating insulin action on amino acid uptake [32] and increasing insulin sensitivity in healthy individuals [22, 42, 43], as well as in individuals with type 2 diabetes [23]. Skeletal muscle exhibits the highest activity of the first two enzymes involved in BCAA degradation [44], and, therefore, is likely to be a major contributor to the reduction in plasma BCAAs caused by acute exercise. However, we cannot exclude that the liver contributes to BCAA disposal directly or indirectly by the uptake of intermediates from the BCAA catabolic pathway released from muscle [44] in order to provide fuel for gluconeogenesis in the fasted state during and shortly after an acute exercise bout.

It is well established that exercise training effectively improves insulin sensitivity in healthy lean individuals, as well as in individuals with obesity and individuals with type 2 diabetes [45–48], and that insulin plays a key role in regulating protein synthesis, particularly in skeletal muscle [4, 5]. However, despite these beneficial effects of exercise training, we showed that neither 10 weeks of endurance training nor 8 weeks of HIIT potentiated the insulin-induced suppression of plasma BCAAs in glucose-tolerant lean individuals, individuals with obesity or individuals with type 2 diabetes. These absent effects of the exercise training interventions on plasma BCAA levels were observed despite marked improvements in whole-body insulin sensitivity, glucose metabolism and glucose clearance in all groups examined. However, our results are in line with previous studies in smaller cohorts, which were also unable to demonstrate an effect of exercise training on the ability of insulin to suppress circulating BCAAs in lean individuals, individuals with obesity or individuals with type 2 diabetes [12, 25].

The underlying causes of elevated circulating BCAAs in obesity and type 2 diabetes remain unclear but may involve reduced mitochondrial BCAA catabolism, as suggested by decreased expression of key enzymes related to BCAA catabolism in skeletal muscle of overweight individuals with dysglycaemia and individuals with type 2 diabetes [31, 49]. Exercise training induces beneficial adaptations in skeletal muscle, including improved mitochondrial function and content, with intact responses in insulin-resistant individuals with obesity and in individuals with type 2 diabetes [16, 17, 50, 51]. In addition, exercise training increases the abundance of proteins involved in BCAA degradation [52], and this response appears to remain intact in overweight individuals with dysglycaemia [31]. However, despite the beneficial effects of exercise training on mitochondrial content and function, several previous studies have reported that exercise training does not affect circulating levels of BCAAs in either healthy lean [24] or overweight individuals [25], or in individuals with type 2 diabetes [12]. In concordance, we demonstrated that exercise training did not reduce fasting plasma BCAAs in individuals with type 2 diabetes, glucose-tolerant individuals with obesity or lean individuals. Taken together, the absent effects of exercise training on fasting plasma BCAAs and the insulin-mediated suppression of plasma BCAAs, despite well-established improvement in insulin sensitivity, suggest that not all insulin-sensitising interventions reduce the potential adverse metabolic effects of elevated circulating levels of BCAAs. This is in contrast with the insulin-sensitising effect of major weight loss, which has been demonstrated to be accompanied by significant reductions in plasma BCAAs [53, 54].

Limitations of our study include the small sample size examined in each sub-study (I–IV), the lack of a healthy lean group in some of the sub-studies (studies II and III) to explore potential abnormalities caused by overweight/obesity per se, as well as the lack of women in the studies involving acute exercise (study II) and exercise training (studies III and IV) in order to rule out sex-specific effects. Even in study I, the number of each sex was too low to perform sex-specific analysis. Moreover, participants, including those with obesity or type 2 diabetes, recruited to exercise studies may be healthier than those in the general population. Another limitation is that the effect of aerobic exercise interventions on plasma BCAAs observed in our studies may differ from the effect of resistance exercise interventions on plasma BCAAs. Although the study participants were instructed not to change their dietary habits during the exercise training periods and to eat a meal of similar size and macronutrient content in the evening before each HEC, we did not register the actual food intake, and, therefore, cannot exclude that potential changes in their food intake could have influenced the results. Methodologically, it would have been advantageous to use specific tracers for the BCAAs to measure their flux and further metabolism; however, our studies were not designed for that originally. Finally, the observational nature of our study makes it difficult to draw conclusions regarding possible mechanisms underlying the impaired insulin-induced suppression of plasma BCAAs and the dissociation between the effects of exercise on insulin sensitivity and plasma BCAAs. Thus, further studies are warranted to identify the cellular and molecular mechanisms explaining the differential effects of exercise training on insulin-stimulated GDR and insulin’s ability to suppress plasma BCAAs, respectively.

In summary, we demonstrated that insulin’s ability to suppress circulating BCAAs is compromised in type 2 diabetes, whereas this response appears intact in obesity. Acute exercise caused a modest reduction in fasting BCAAs, and this response was preserved in type 2 diabetes. However, this effect of acute exercise did not potentiate insulin-induced suppression of plasma BCAAs. Furthermore, exercise training did not reduce fasting BCAAs despite improving insulin sensitivity, nor did it influence insulin’s ability to suppress plasma BCAAs. While the impaired insulin suppression of plasma BCAAs in type 2 diabetes supports the notion that insulin resistance contributes to elevated plasma BCAAs in the fasting, postabsorptive state, the inability of acute exercise and exercise training to potentiate insulin-mediated suppression of plasma BCAAs suggests a more complex relationship between insulin sensitivity and BCAAs under some circumstances. Further studies are warranted to disentangle the causal mechanisms underlying the relationship between insulin resistance and elevated plasma BCAAs in humans.

## Supplementary Information

Below is the link to the electronic supplementary material.

ESM (PDF 888 KB)

## Data Availability

The data presented in this study are not available to the public. However, researchers with the appropriate legal permissions from the Danish Data Protection Agency may request access. Inquiries about accessing the datasets should be sent to the corresponding author.

## Abbreviations

BCAA: Branched-chain amino acid
GDR: Glucose disposal rate
HEC: Hyperinsulinaemic–euglycaemic clamp
HIIT: High-intensity interval training
MCR: Metabolic clearance rate

## Authors’ relationship and activities

First author, PMM, owns personal stocks in Novo Nordisk A/S. JFPW holds shares in Pfizer Inc. and Novo Nordisk A/S. JFPW is currently a consultant at Pfizer Inc. The authors declare that there are no other relationships or activities that might bias, or be perceived to bias, their work.
