# Correction: Effect of acute exercise and exercise training on the ability of insulin to clear branched‑chain amino acids from plasma in obesity and type 2 diabetes

**DOI:** 10.1007/s00125-026-06690-w

**Published:** 2026-03-19

**Authors:** Pauline M. Møller, Rasmus Kjøbsted, Maria H. Petersen, Martin E. de Almeida, Andreas J. T. Pedersen, Jørgen F. P. Wojtaszewski, Kurt Højlund

**Affiliations:** 1 Steno Diabetes Center Odense, Odense University Hospital, Odense, Denmark; 2 Department of Clinical Research, University of Southern Denmark, Odense, Denmark; 3 The August Krogh Section for Molecular Physiology, Department of Nutrition, Exercise and Sports, University of Copenhagen, Copenhagen, Denmark; 4 Department of Sports Science and Clinical Biomechanics, University of Southern Denmark, Odense, Denmark

**Correction: Diabetologia**

10.1007/s00125-025-06454-y

The units for plasma branched‑chain amino acid concentrations were incorrectly reported as ‘pmol/l’ throughout the manuscript, figures and electronic supplementary material. The correct unit is ‘µmol/l’. This typographical error does not affect the reported numeric values, statistical analyses or the conclusions of the paper. The original article has been corrected.

**Fig. 1:**
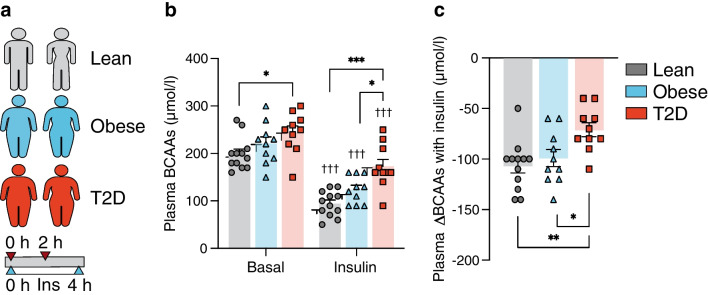
Study I: plasma BCAAs were measured (**a**) before (0 h) and after (2 h) insulin infusion during a 4 h HEC in 12 glucose-tolerant lean participants, ten glucose-tolerant individuals with obesity and ten individuals with type 2 diabetes. (**b**) Plasma BCAAs in the basal and insulin-stimulated states in the lean (grey), obese (blue) and type 2 diabetes (orange) groups. (**c**) Insulin-mediated changes in plasma BCAAs (ΔBCAAs) in the lean (grey), obese (blue) and type 2 diabetes (orange) groups. The data are presented as means ± SEM. *p<0.05, **p<0.01, ***p<0.001 as indicated, †††*p*<0.001 vs basal. Ins, insulin; T2D, type 2 diabetes

**Fig. 2:**
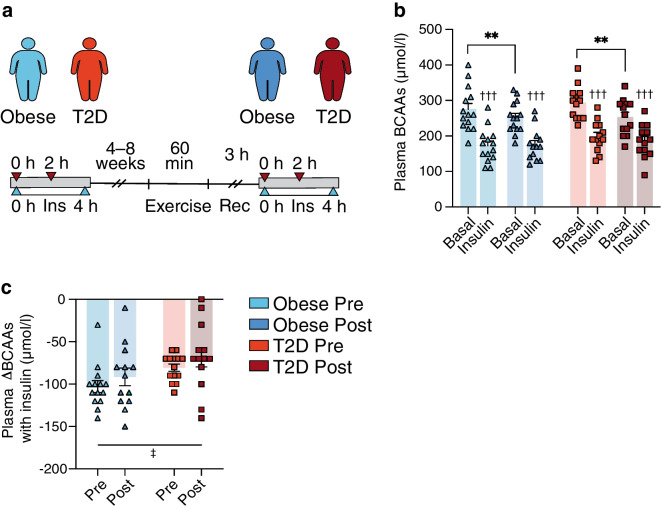
Study II: plasma BCAAs were measured (**a**) before (0 h) and after (2 h) insulin infusion during two 4 h HECs in 14 glucose-tolerant individuals with obesity and 13 individuals with type 2 diabetes performed 4–8 weeks prior to an acute bout of exercise (60 min at 70% of $\dot{V}{\mathrm{O}}_{2\mathrm{max}}$) and again 3 h into recovery. (**b**) Plasma BCAAs prior to exercise in the obese (light blue) and type 2 diabetes (orange) groups and following exercise in the obese (dark blue) and type 2 diabetes (red) groups, both in the basal and insulin-stimulated states. (**c**) Insulin-mediated changes in plasma BCAAs (ΔBCAAs) in the obese (light blue) and type 2 diabetes (orange) groups before exercise (Pre) and in the obese (dark blue) and type 2 diabetes (red) groups following exercise (Post). The data are shown as means ± SEM. **p<0.01 as indicated, †††*p*<0.001 vs basal, ‡*p*<0.05 main effect of groups. Ins, insulin; Rec, recovery; T2D, type 2 diabetes

**Fig. 3:**
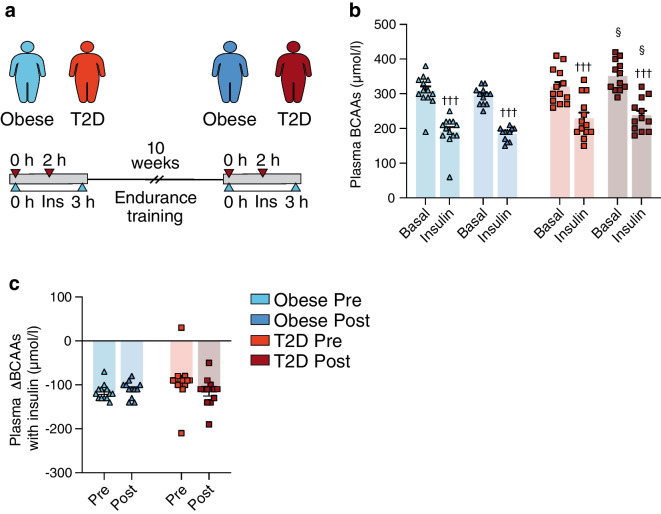
Study III: plasma BCAAs were measured (**a**) before (0 h) and after (2 h) insulin infusion during two 3 h HECs in 13 glucose-tolerant individuals with obesity and 13 individuals with type 2 diabetes performed 1–2 weeks before (Pre) and 48 h after (Post) 10 weeks of endurance exercise training. (**b**) Plasma BCAAs before exercise training in the obese (light blue) and type 2 diabetes (orange) groups and following exercise training in the obese (dark blue) and type 2 diabetes (red) groups, both in the basal and insulin-stimulated states. (**c**) Insulin-mediated changes in plasma BCAAs (ΔBCAAs) in the obese (light blue) and type 2 diabetes (orange) groups before and in the obese (dark blue) and type 2 diabetes (red) groups following exercise training. The data are shown as means ± SEM. †††*p*<0.001 vs basal, §*p*<0.05 vs Obese Post. T2D, type 2 diabetes

**Fig. 4:**
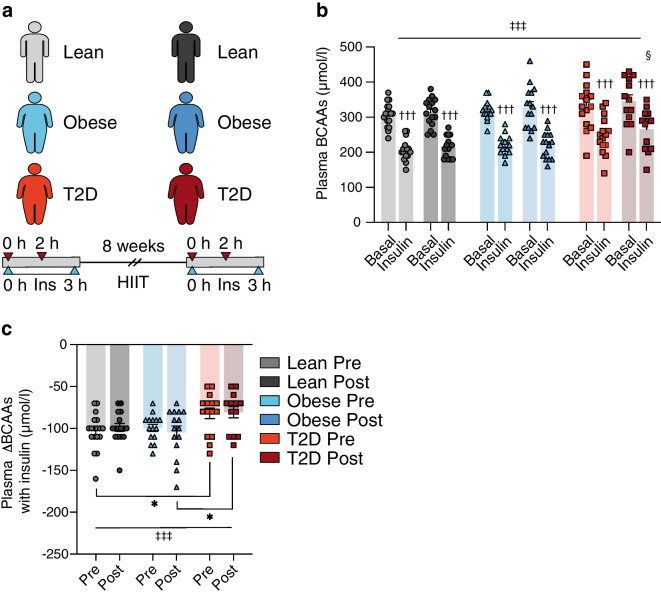
Study IV: plasma BCAAs were measured (**a**) before (0 h) and after (2 h) insulin infusion during two 3 h HECs in 18 glucose-tolerant lean individuals, 15 glucose-tolerant individuals with obesity and 15 individuals with type 2 diabetes performed 1–2 weeks before (Pre) and 48 h after (Post) 8 weeks of HIIT. (**b**) Plasma BCAAs before HIIT in the lean (light grey), obese (light blue) and type 2 diabetes (orange) groups and after HIIT in the lean (dark grey), obese (dark blue) and type 2 diabetes (red) groups, both in the basal and insulin-stimulated states. (**c**) Insulin-mediated changes in plasma BCAAs (ΔBCAAs) in the lean (light grey), obese (light blue) and type 2 diabetes (orange) groups before HIIT and in the lean (dark grey), obese (dark blue) and type 2 diabetes (red) groups after HIIT. The data are shown as means ± SEM. *p<0.05 as indicated, †††*p*<0.001 vs basal, ‡‡‡*p*<0.001 main effect of group, §*p*<0.05 vs Lean Post. T2D, type 2 diabetes

