# Supplementary material for: Effect of acute exercise and exercise training on the ability of insulin to clear branched-chain amino acids from plasma in obesity and type 2 diabetes
Source: Diabetologia. 2025 May 22;68(8):1789–800. doi: 10.1007/s00125-025-06454-y (PMC12246017; doi:10.1007/s00125-025-06454-y)
Supplement: Supplementary file 1 — ESM (PDF 888 KB) [file 125_2025_6454_MOESM1_ESM.pdf]

Electronic Supplementary Material

ESM Tables

ESM Table 1.
Clinical and metabolic characteristics (Study I)

	**Lean controls**	**Obese controls**	**T2D**
n	12	10	10
**Sex (m/f)**	6/6	6/4	5/5
**Age (years)**	54.5 ± 1.3	55.4 ± 1.2	53.9 ± 1.6
**BMI (kg/m^2)**	23.4 ± 0.5	31.1 ± 0.9***	29.8 ± 1.3***
**Lean body mass (kg)**	53.5 ± 3.7	63.0 ± 4.2	54.2 ± 3.2
**Fat mass (kg)**	17.6 ± 0.9	30.3 ± 1.9**	29.6 ± 2.9**
**Fasting plasma glucose (mmol/l)**	5.5 ± 0.1	5.8 ± 0.1	8.9 ± 0.7††***
**Fasting serum insulin (pmol/l)**	30 ± 4	40 ± 6	85 ± 16†***
**HbA$_{1c}$ (%)**	5.5 ± 0.1	5.3 ± 0.1	6.8 ± 0.4†††***
**Fasting plasma cholesterol (mmol/l)**	5.6 ± 0.2	5.1 ± 0.3	4.9 ± 0.2
**Fasting plasma triacylglycerol (mmol/l)**	0.9 ± 0.1	1.0 ± 0.1	1.6 ± 0.2†††**
**GDR, clamp (mg/min/m^2)***	380 ± 25	309 ± 24*	160 ± 26†††***
**MCR glucose, clamp (l/min/m^2)***	38.6 ± 3.0	31.3 ± 2.3*	16.8 ± 2.9†††***

Data are mean ± SEM. $^{*}p<0.05$, $^{**}p<0.01$ and $^{***}p<0.001$ vs. lean controls, $^{\dagger}p<0.05$, $^{\dagger\dagger}p<0.01$ and $^{\dagger\dagger\dagger}p<0.001$ vs. obese controls. *From the hyperinsulinemic-euglycemic clamp in study I. Statistical analyses were performed as reported [34, 35]. GDR, glucose disposal rate; MCR, metabolic clearance rate; T2D, Type 2 diabetes. Data reproduced from [34, 35] with permission from Springer Nature.

ESM Table 2.
Clinical and metabolic characteristics with and without prior acute exercise (Study II)

	Obese controls	T2D
n	14	13
**Age (years)**	55 ± 2	55 ± 2
**BMI (kg/m^2)**	29.0 ± 0.9	29.7 ± 1.0
**Lean body mass (kg)**	69.1 ± 2.4	68.3 ± 2.0
**Fat mass (kg)**	24.5 ± 1.9	28.1 ± 2.4
**Fasting plasma glucose (mmol/l)**	5.6 ± 0.1	10.0 ± 0.7***
**Fasting serum insulin (pmol/l)**	40 ± 5	56 ± 12
**HbA$_{1c}$ (%)**	5.5 ± 0.1	7.0 ± 0.2***
**HbA$_{1c}$ (mmol/mol)**	37 ± 4.0	53 ± 7.9***
**Fasting plasma cholesterol (mmol/l)**	5.7 ± 0.3	4.4 ± 0.4*
**Fasting plasma triacylglycerol (mmol/l)**	1.5 ± 0.2	3.2 ± 1.5
**VO$_{2max}$ (ml/min/kg)**	37.8 ± 2.01	33.6 ± 2.35
**GDR clamp – without prior exercise (mg/min/m^2)**	349 ± 35	242 ± 35**
**GDR clamp – with prior exercise (mg/min/m^2)**	333 ± 28	245 ± 28**
**MCR glucose, clamp – without prior exercise (l/min/m^2)**	35.3 ± 3.4	25.3 ± 3.7*
**MCR glucose, clamp – with prior exercise (l/min/m^2)**	34.5 ± 2.9	25.2 ± 2.8*

Data are means ± SEM. *p<0.05, **p<0.01 and ***p<0.001 vs. controls. Statistical analyses were performed as reported [33]. GDR, glucose disposal rate; MCR, metabolic clearance rate; T2D, Type 2 diabetes. Data reproduced from [33] with permission from Springer Nature.

ESM Table 3.
Clinical and metabolic characteristics before and after 10 weeks endurance training (Study III)

	**Obese controls**	**T2D**
***n* (before/after training)**	13/12	13/12
**Age (years)**	52.7 ± 1.6	52.8 ± 1.3
**Fasting plasma glucose (mmol/l)**	5.8 ± 0.1	9.4 ± 0.5***
**Fasting serum insulin (pmol/l)**	64 ± 7	82 ± 11
**HbA_{1c} (%)**	5.2 ± 0.1	7.2 ± 0.3***
**Fasting plasma cholesterol (mmol/l)**	5.5 ± 0.2	5.0 ± 0.2
**Fasting plasma triacylglycerol (mmol/l)**	1.69 ± 0.17	2.37 ± 0.32
**BMI – before training (kg/m^2)**	33.0 ± 0.7	33.5 ± 0.1
**BMI – after training (kg/m^2)**	32.7 ± 0.8†††	33.4 ± 0.1
**Lean body mass – before training (kg)**	74.7 ± 2.1	72.7 ± 1.9
**Lean body mass – after training (kg)**	75.9 ± 2.2	74.3 ± 1.7
**Fat mass – before training (kg)**	35.7 ± 2.5	34.9 ± 2.1
**Fat mass – after training (kg)**	32.9 ± 2.5	33.7 ± 1.9
**VO_{2max} – before training (ml/min/kg)***	27.8 ± 1.5	26.7 ± 0.9
**VO_{2max} – after training (ml/min/kg)***	34.6 ± 2.2†††	29.8 ± 1.2†††
**GDR clamp – before training (mg/min/m^2)**	327 ± 22	208 ± 26***
**GDR clamp – after training (mg/min/m^2)**	408 ± 38†††	242 ± 30***†††
**MCR glucose, clamp – before training (l/min/m^2)**	34.4 ± 2.2	22.1 ± 2.8**
**MCR glucose, clamp – after training (l/min/m^2)**	42.2 ± 4.1†	25.3 ± 3.4**†

Data are means ± SEM. *p<0.05, **p<0.01 and ***p<0.001 vs. controls. †p<0.05 and †††p<0.001 vs. pre-training. *n=10 type 2 diabetes patients after training. Statistical analyses were performed as reported [15]. GDR, glucose disposal rate; MCR, metabolic clearance rate; T2D, Type 2 diabetes. Data reproduced from [15] with permission from Springer Nature.

ESM Table 4.
Clinical and biochemical characteristics before and after HIIT (Study IV)

	Lean controls	Obese controls	T2D
***n* (before after HIIT)**	18/16	15/15	15/13
**Age (years)**	56.2±1.5	53.8±1.8	55.2±1.7
**Fasting plasma glucose (mmol/l)**	5.2±0.1	5.6±0.1	9.6±0.7**††
**Fasting serum insulin (pmol/l)**	61±9	71±8	117±19*†
**HbA_{1c} (mmol/mol)**	35±1	35±1	54±4**††
**HbA_{1c} (%)**	5.4±0.1	5.3±0.1	7.1±0.3**††
**Fasting plasma cholesterol (mmol/l)**	4.9±0.2	5.4±0.1	4.9±0.3
**Fasting plasma triacylglycerol (mmol/l)**	1.52±0.23	1.59±0.19	2.47±0.40*†
**BMI – before HIIT (kg/m^2)**	24.0±0.4	30.8±0.7**	31.2±0.8**
**BMI – after HIIT (kg/m^2)**	23.7±0.4‡	30.3±0.6‡**	30.8±0.9‡**
**Lean body mass – before HIIT (kg)**	56.9±1.3	65.3±1.3**	64.8±1.7**
**Lean body mass – after HIIT (kg)**	57.1±1.4‡	66.2±1.2‡**	66.8±2.0‡**
**Fat mass – before HIIT (kg)**	20.1±1.0	32.0±1.9**	34.8±2.3**
**Fat mass – after HIIT (kg)**	18.2±1.2‡‡	29.7±1.8‡‡**	33.0±2.5‡‡**
**VO_{2max} – before HIIT (ml/kg/min)**	38.0±1.5	33.4±1.8*	25.8±0.9**††
**VO_{2max} – after HIIT (ml/kg/min)**	42.8±1.7‡‡	36.6±1.2‡*	30.5±1.0‡‡**†
**GDR clamp – before HIIT (mg/min/m^2)**	356±30	351±26	210±24*†
**GDR clamp – after HIIT (mg/min/m^2)**	463±36‡‡	447±29‡	317±36‡‡*†
**MCR glucose, clamp – before HIIT (l/min/m^2)**	36.5 ± 3.1	37.9 ± 2.9‡	21.7 ± 2.5*††
**MCR glucose, clamp – after HIIT (l/min/m^2)**	50.2 ± 4.4	45.8 ± 2.9‡	34.2 ± 3.8‡‡*†

Data are means ± SEM. *p<0.05 and **p<0.001 vs. lean controls, †p<0.05 and ††p<0.001 vs. obese controls, ‡*p*<0.05 and ‡‡*p*<0.001 vs. before HIIT. Statistical analyses were performed as reported [20]. GDR, glucose disposal rate; HIIT, High intensity interval training; MCR, metabolic clearance rate; T2D, Type 2 diabetes. Data reproduced from [20] under a CC BY license.

ESM Figures

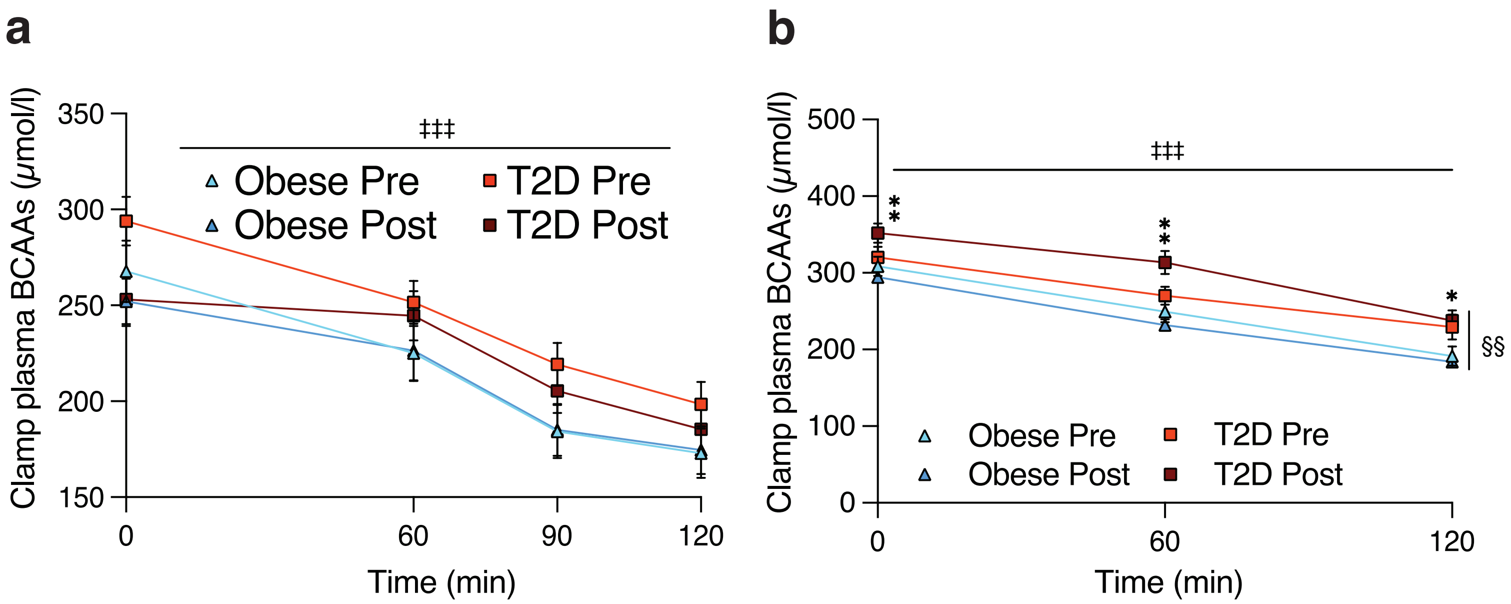

ESM Fig. 1 Plasma branched-chain amino acids (BCAAs) measured (**a**) from study II during a hyperinsulinemic-euglycemic clamp at basal (0 min) and after 60, 90, and 120 min insulin infusion in 13 glucose-tolerant obese patients (blue, triangle) and 13 patients with type 2 diabetes (T2D) (orange, square) 4-8 weeks prior to (Pre) acute exercise and again in the obese (dark blue, triangle) and T2D (red, square) groups 3 h into recovery after (Post) acute exercise. Plasma BCAA concentrations measured (**b**) from study III during a hyperinsulinemic-euglycemic clamp at basal (0 min) and after 60 min and 120 min insulin infusion in 13 glucose-tolerant obese patients (blue, triangle) and in 13 patients with T2D (orange, square) before (Pre) 10-weeks of endurance exercise training and in the obese (dark blue, triangle) and T2D (red, square) groups 48 h after (Post) 10 weeks of exercise training. The data are presented as means ± SEM. *$p<0.05$ and **$p<0.01$ T2D Post vs. Obese Post, ‡‡‡$p<0.001$ main effect of time, and §§$p<0.001$ main effect of groups.
